# Mercaptopurine for the Treatment of Ulcerative Colitis: A Randomized Placebo-Controlled Trial

**DOI:** 10.1093/ecco-jcc/jjad022

**Published:** 2023-02-27

**Authors:** Mark Löwenberg, Adriaan Volkers, Sara van Gennep, Aart Mookhoek, Nahid Montazeri, Esmé Clasquin, Marjolijn Duijvestein, Adriaan van Bodegraven, Svend Rietdijk, Jeroen Jansen, Dirk van Asseldonk, Esmerij van der Zanden, Marcel Dijkgraaf, Rachel West, Nanne de Boer, Geert D’Haens

**Affiliations:** Department of Gastroenterology and Hepatology, Amsterdam University Medical Center, Amsterdam, the Netherlands; Department of Gastroenterology and Hepatology, Amsterdam University Medical Center, Amsterdam, the Netherlands; Department of Gastroenterology and Hepatology, Amsterdam University Medical Center, Amsterdam, the Netherlands; Institute of Pathology, University of Bern, Bern, Switzerland; Biostatistics Unit, Department of Gastroenterology and Hepatology, Amsterdam University Medical Center, Amsterdam, the Netherlands; Department of Gastroenterology and Hepatology, Amsterdam University Medical Center, Amsterdam, the Netherlands; Department of Gastroenterology and Hepatology, RadboudUMC, Nijmegen, the Netherlands; Department of Gastroenterology and Hepatology, AGEM Research Institute, Amsterdam University Medical Center, Vrije Universiteit Amsterdam, Amsterdam, the Netherlands; Department of Gastroenterology, Geriatrics, Internal and Intensive Care Medicine (Co-MIK), Zuyderland Medical Centre, Sittard-Geleen/Heerlen, the Netherlands; Department of Gastroenterology and Hepatology, OLVG, Amsterdam, the Netherlands; Department of Gastroenterology and Hepatology, OLVG, Amsterdam, the Netherlands; Department of Gastroenterology and Hepatology, Noordwest Ziekenhuisgroep, Alkmaar, the Netherlands; Department of Gastroenterology and Hepatology, Amstelland Ziekenhuis, Amstelveen, the Netherlands; Department of Epidemiology and Data Science, University Medical Center, Amsterdam, the Netherlands; Department of Gastroenterology and Hepatology, Franciscus Gasthuis, Rotterdam, the Netherlands; Department of Gastroenterology and Hepatology, AGEM Research Institute, Amsterdam University Medical Center, Vrije Universiteit Amsterdam, Amsterdam, the Netherlands; Department of Gastroenterology and Hepatology, Amsterdam University Medical Center, Amsterdam, the Netherlands

**Keywords:** Ulcerative colitis, immunomodulators, therapeutic drug monitoring, randomized controlled trial

## Abstract

**Background and Aims:**

Scepticism about the efficacy of thiopurines for ulcerative colitis [UC] is rising. This study aimed to evaluate mercaptopurine treatment for UC.

**Methods:**

In this prospective, randomized, double-blind, placebo-controlled trial, patients with active UC, despite treatment with 5-aminosalicylates [5-ASA], were randomized for therapeutic drug monitoring [TDM]-guided mercaptopurine treatment or placebo for 52 weeks. Corticosteroids were given in the first 8 weeks and 5-ASA was continued. Proactive metabolite-based mercaptopurine and placebo dose adjustments were applied from week 6 onwards by unblinded clinicians. The primary endpoint was corticosteroid-free clinical remission and endoscopic improvement [total Mayo score ≤2 points and no item >1] at week 52 in an intention-to-treat analysis.

**Results:**

Between December 2016 and April 2021, 70 patients were screened and 59 were randomized at six centres. In the mercaptopurine group, 16/29 [55.2%] patients completed the 52-week study, compared to 13/30 [43.3%] on placebo. The primary endpoint was achieved by 14/29 [48.3%] patients on mercaptopurine and 3/30 [10%] receiving placebo (Δ = 38.3%, 95% confidence interval [CI] 17.1–59.4, *p* = 0.002). Adverse events occurred more frequently with mercaptopurine [808.8 per 100 patient-years] compared to placebo [501.4 per 100 patient-years]. Five serious adverse events occurred, four on mercaptopurine and one on placebo. TDM-based dose adjustments were executed in 22/29 [75.9%] patients, leading to lower mercaptopurine doses at week 52 compared to baseline.

**Conclusions:**

Optimized mercaptopurine treatment was superior to placebo in achieving clinical, endoscopic and histological outcomes at 1 year following corticosteroid induction treatment in UC patients. More adverse events occurred in the mercaptopurine group.

## Graphical Abstract

**Graphical Abstract:**
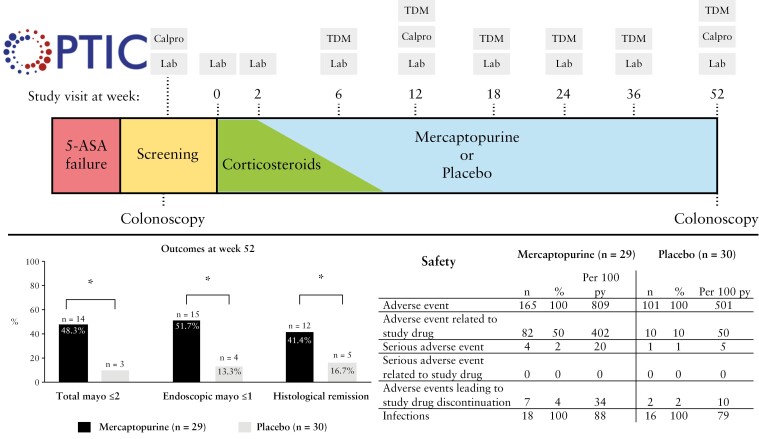

## 1. Introduction

Thiopurines have been used for the treatment of ulcerative colitis [UC] since the early 1960s.^1^ It is estimated that ~25% of UC patients receive treatment with thiopurines.^2^ However, up to 40% of patients have to discontinue thiopurines due to adverse events.^3,4^ Literature reviews have concluded that maintenance treatment with thiopurines is efficacious in UC, but also stated that controlled studies were of relatively low quality.^5–7^ In these studies, patients were often not blinded to treatment allocation, and dosing of thiopurines was based solely on body weight without the use of therapeutic drug monitoring [TDM].^8–13^ TDM holds that concentrations of thiopurine metabolites 6-thioguaninenucleotides [6-TGN] and 6-methylmercaptopurine [6-MMP] are measured in the red blood cells [RBCs] and thiopurine doses are adjusted based on those concentrations. The Lennard and the more user-friendly Dervieux method are the most widely used methods to measure 6-TGN and 6-MMP RBC concentrations.^14^ Thiopurine metabolism varies highly amongst patients and there is no clear dose–response association.^15^ However, therapeutic 6-TGN RBC concentrations are associated with improved clinical efficacy and high 6-TGN or 6-MMP RBC concentrations are associated with toxicity.^15,16^ Therefore, TDM provides an opportunity to improve efficacy and reduce the number of side effects of thiopurines. An earlier trial found a trend towards improved efficacy of TDM-based azathioprine vs weight-based azathioprine for Crohn’s disease.^17^ However, this difference was not significant, probably due to the small sample size. Thiopurine-treated patients with a so-called skewed metabolism can develop high 6-MMP concentrations. Adding allopurinol co-medication is beneficial for these patients.^18^ Proactive TDM-optimized use of thiopurines may lead to prolonged disease control, thereby avoiding costly treatment intensification or surgery.^19,20^ Therefore, TDM-based dosing is considered as the optimal treatment strategy for UC patients receiving thiopurines.

OPTIC [OPtimised Thiopurines In ulcerative Colitis] aimed to investigate the efficacy of optimized thiopurine treatment compared to placebo for UC in a prospective placebo-controlled trial using objective outcome measures.

## 2. Materials and Methods

### 2.1 Study population

Adult patients [between 18 and 80 years] with a confirmed diagnosis of UC were enrolled at six hospitals in the Netherlands [two academic and four non-academic teaching hospitals]. Eligible patients had an indication to start oral prednisone or budesonide treatment, based on clinical and endoscopic signs of active UC, despite daily use of ≥2 g oral 5-aminosalicylates [5-ASA]. Patients with previous exposure to thiopurines or biologic agents were excluded. Pregnant patients were excluded, as well as patients with known chronic obstructive pulmonary disease, acute coronary heart disease, active malignancy, a history of high-grade colonic dysplasia or colonic cancer, previous [subtotal] colectomy, concomitant medication use interfering with mercaptopurine metabolism, gastric ulcers or active substance misuse. Additional exclusion criteria were a positive tuberculosis screening test, active hepatitis B or C infection, leukopaenia [leukocyte count <1.8 × 10^9^/L], thrombocytopaenia [thrombocyte count <90 × 10^9^/L], abnormal renal function [estimated glomerular filtration rate <30 mL/min] or any other condition which could interfere with the subject’s ability to comply with the study procedures. Prior to enrolment, infectious colitis was excluded. All authors had access to the study data and reviewed and approved the final manuscript. This trial was registered as EudraCT: 2015-005260-41.

### 2.2 Study design

**Figure 1.**
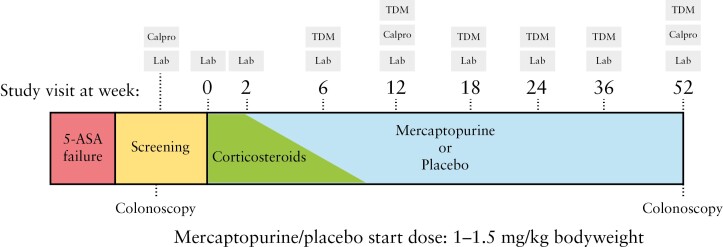
Graphical study-design. 5-ASA = 5-aminosalicylates, Calpro = faecal calprotectin test, Lab = laboratory blood tests, TDM = therapeutic drug monitoring.

This was a prospective, multicentre, double-blind, randomized, placebo-controlled study on mercaptopurine treatment for UC with a follow-up period of 52 weeks [Figure 1]. Patients signed informed consent prior to screening. Screening consisted of a complete colonoscopy or sigmoidoscopy, assessment of clinical and endoscopic disease activity with the full 3-day Mayo^21^ score and ulcerative colitis endoscopic index of severity [UCEIS],^22^ routine laboratory testing, faecal calprotectin, and stool tests [including *Clostridium difficile* toxins, *Salmonella*, *Shigella*, *Yersinia*, *Campylobacter* and, if indicated, parasites]. Genetic thiopurine *S*-methyltransferase [TPMT] polymorphism analysis was not part of the study protocol, as this is not routinely performed in Dutch clinical practices. Moreover, TDM-based dose adjustments allowed participants to reach a therapeutic thiopurine metabolite level, also in participants with TPMT polymorphisms. At the same time, TDM allowed testing for drug compliance. All participants received remission induction treatment with 9 mg budesonide [Cortiment] per day for 8 weeks [without tapering] or a prednisone tapering scheme [40 mg/day for 2 weeks, then 30 mg/day for 1 week, followed by a weekly dose decrease of 5 mg/day]. Induction treatment with budesonide and prednisone could be prolonged at the physician’s discretion, but had to be discontinued before week 12. All patients continued concomitant oral 5-ASA treatment during the study, except in case of intolerance.

Participants were randomized in a 1:1 ratio using variable block randomization with a maximum block size of eight. Randomization was stratified for prednisone or budesonide treatment. Patients and physicians were blinded to treatment allocation. After inclusion, the site investigator requested randomization via the online electronic case report forms [eCRF] website [Castor EDC] using certified randomization software. Subsequently, an unblinded research coordinator [EC] performed the randomization centrally. After randomization, the unblinded research coordinator sent the results to the trial pharmacy of the Amsterdam UMC, location Academic Medical Center [AMC], which provided the study medication.

Participants underwent complete colonoscopy or sigmoidoscopy with mucosal biopsies of the most severely affected mucosa at screening and at week 52. Biopsies were taken from the rectum when no endoscopic disease activity was observed. All procedures were videotaped and assessed using the endoscopic Mayo score by a central reader [ML], who was blinded to treatment assignments and clinical information. A blinded pathologist [AM] assessed histological disease activity using the Geboes score, Robarts histopathology index and Nancy score. Faecal calprotectin levels were measured at screening, week 12 and week 52. Laboratory measurements were performed at baseline and weeks 2, 6, 12, 18, 24, 36 and 52. TDM was applied simultaneously, starting from week 6 onwards. Laboratory measurements included haemoglobin, haematocrit, mean corpuscular volume [MCV], red and white blood cell count, white blood cell differentiation, platelet count, creatinine, C-reactive protein [CRP], albumin, aspartate aminotransferase [AST], alanine transaminase [ALT], alkaline phosphatase, gamma-glutamyl transferase and lipase. Adverse events were documented throughout the study and were graded as mild, moderate or severe at the physician’s discretion. Infections were classified as mild [no oral antibiotics or antiviral medication required], moderate [requiring oral antibiotics or antiviral medication] or severe [requiring intravenous treatment or hospitalization]. In case of early discontinuation of the study drug, adverse events were documented until 30 days after the last dose. A possible relationship of the adverse event with mercaptopurine or placebo was based on the physician’s discretion. In case of a worsening of UC, 5-ASA treatment could be optimized, and an additional mercaptopurine TDM could be performed. A ‘flare visit’ was performed if suspicion of active UC persisted. At this visit, infectious stool and faecal calprotectin tests were taken, blood analysis was performed and, if appropriate, an additional endoscopy was performed. Patients could continue the study drug if endoscopic and clinical disease activity were absent or had sufficiently improved, according to the physician’s discretion. Patients were considered a treatment failure when clinical and endoscopic active disease was confirmed, and these were treated at the physician’s discretion thereafter.

The medical ethics committee of the Amsterdam UMC, location AMC, Amsterdam, the Netherlands, approved this study. Data were collected in an online eCRF [Castor EDC]. A trial monitor performed data source verification at each study site.

### 2.3 TDM and mercaptopurine/placebo dose adjustments

The initial mercaptopurine or placebo dose was 25 mg/day for the first week, followed by an increase to 1–1.5 mg/kg body weight, according to the ECCO guideline.^23^ 6-TGN and 6-MMP RBC concentrations were measured at weeks 6, 12, 18, 24, 36 and 52 using the Dervieux method.^14^ If inefficacy or an adverse event occurred, 6-TGN and 6-MMP RBC concentrations could be assessed an extra time and dose adjustments could be applied by the unblinded clinicians. Patients and physicians were blinded for 6-TGN and 6-MMP results. Two unblinded clinicians [AvB and MDu] received the results and communicated TDM-based dose or decreases to the blinded study physicians using a predefined dosing algorithm [Supplementary Figure 1]. The target 6-TGN concentration was 600–1200 pmol/8 × 10^8^ RBC [corresponding to 230–460 pmol/8 × 10^8^ RBC if the Lennard method is used] and 6-MMP <5700 pmol/8 × 10^8^ RBC.^14,24^ If 6-MMP exceeded 5700 pmol/8 × 10^8^ RBC or if 6-TGN was <300 pmol/8 × 10^8^ RBC with a 6-MMP/6-TGN ratio >10, the unblinded clinicians advised to start 100 mg allopurinol per day and reduce the mercaptopurine dose to 25–33% of the previous dose. To mimic TDM, every placebo patient was randomized a second time to a predefined dose adjustment scheme. Patients in the placebo group were never instructed to start allopurinol.

Placebo or mercaptopurine dose adjustments were also made by study physicians or unblinded clinicians based on intolerance and adverse events as well as laboratory results [Supplementary Figure 2]. In case of gastrointestinal intolerance, leukopaenia [leukocyte count <3 × 10^9^/L], thrombocytopaenia [thrombocyte count <50 × 10^9^/L], or AST, or ALT >3× the upper limit of normal [ULN], mercaptopurine or placebo was discontinued for 3–14 days. The study medication was re-initiated with 25 mg/day when symptoms had resolved. Mercaptopurine or placebo was permanently discontinued in case of pancreatitis [confirmed clinically, biochemically and/or at imaging], hepatotoxicity [ALT or AST >8× ULN], severe leukopaenia [leukocyte count <0.5 × 10^9^/L] or thrombocytopaenia [thrombocyte count <25 × 10^9^/L] or, if leukopaenia, thrombocytopenia or another study drug-related adverse events did not recover.

### 2.4 Endpoints and definitions

The primary endpoint was corticosteroid-free combined clinical remission and endoscopic improvement at week 52 [i.e. ≤2 points, and no item >1, using the 12-point Mayo score consisting of stool frequency, rectal bleeding, endoscopic score and the physician’s global assessment].^21^ Additionally, a per-protocol analysis was done for patients who completed the 52-week follow-up period and reached the primary endpoint. Secondary endpoints were corticosteroid-free endoscopic improvement [i.e. endoscopic Mayo score = 0 or 1], clinical remission [i.e. Mayo rectal bleeding score = 0 and Mayo stool frequency score = 0 or 1] and histological remission [i.e. absence of neutrophils in the mucosa; Geboes score <2 B.1, Robarts histopathology index ≤3 and/or Nancy score ≤1], at week 52. Other endpoints included combined clinical and endoscopic response [i.e. 3-point and 30% reduction compared to baseline and 1-point drop in the rectal bleeding score or a rectal bleeding score ≤1], clinical response [i.e. ≥2-point drop in the 6-point Mayo score, consisting of rectal bleeding and stool frequency items, compared to baseline] and biochemical remission [i.e. CRP <5 mg/L and faecal calprotectin <250 mg/kg] at week 52. In addition, the proportions of patients below several UCEIS cut-offs at week 52 were calculated. A safety analysis was performed on adverse events that occurred during the study.

### 2.5 Sample size calculation and statistical methods

A two-group chi-squared test with a 5% two-sided significance level had 80% power to detect a difference between a group 1 proportion, π_1_, of 0.15 [placebo] and a group 2 proportion, π_2_, of 0.35 [mercaptopurine] [odds ratio of 3.051] when the sample size in each group is 73. Considering a 5% possible drop-out, this resulted in a sample size of 154 participants. This power calculation was made using nQuery [nQuery Sample Size Software version 8.5.1, Statsols].

Descriptive statistics were described as proportions with percentages or as means with standard deviations [SD] unless stated otherwise. Results were analysed according to an intention-to-treat principle, including all patients who received at least one dose of mercaptopurine or placebo. Participants who discontinued study treatment before week 52 or were lost to follow-up were considered non-responders [non-responder imputation]. Proportions were compared with a chi-square or Fischer’s exact test, if appropriate. To reduce the risk for multiple testing on this cohort, a hierarchical testing order was applied to a limited number of endpoints. If a test resulted in a significant result, the subsequent comparison could be performed. The hierarchical order was [I] combined clinical remission and endoscopic improvement, [II] endoscopic improvement, [III] clinical remission and [IV] histological remission. Other endpoints were presented as proportions without statistical comparisons. Unpaired continuous variables were non-parametrically compared with the Mann–Whitney U test, and the Wilcoxon signed-rank test was used for paired non-parametric comparisons. A *p*-value <0.05 was considered significant. Statistical analyses were performed using SPSS [SPSS version 28.0, IBM].

## 3. Results

### 3.1 Baseline characteristics

**Figure 2.**
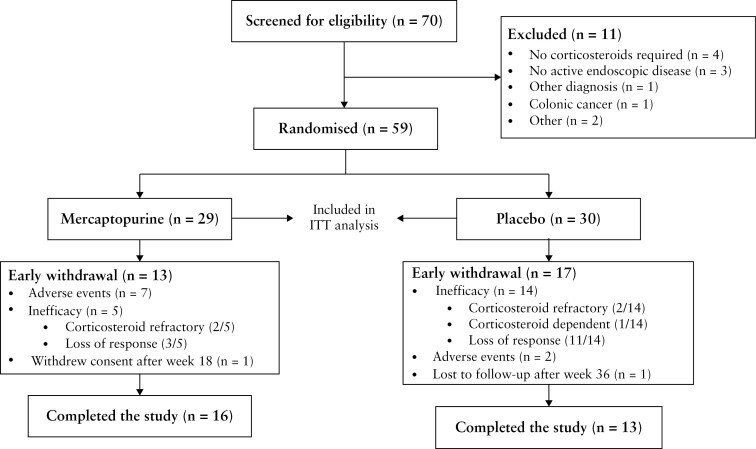
Flowchart. ITT = intention to treat.

Between December 2016 and April 2021, 70 patients were screened for eligibility. In total, 29 and 30 patients were randomized to receive mercaptopurine and placebo, respectively [Figure 2]. Due to slow recruitment, the steering committee and sponsor decided to stop inclusion prematurely. The last patient visit took place in April 2022. All 59 patients took at least one dose of the study drug and were included in the intention-to-treat analysis.

Baseline characteristics were comparable between the two groups [Table 1]. The mean disease duration of the mercaptopurine group at baseline was 8.7 years [SD 9.4] compared to 6.6 years [SD 7.1] in the placebo group. The placebo group contained three [10%] smokers, while none of the patients in the mercaptopurine group were active smokers. Both groups were comparable in terms of induction treatment regimens, disease extension and endoscopic disease severity.

**Table 1.** Baseline characteristics

	Mercaptopurine	Placebo
Male/female	18/11	19/11
Age, years, mean [SD]	43.4 [15.6]	41.5 [13.3]
Age at diagnosis, years, mean [SD]	34.7 [10.7]	34.9 [12.0]
Disease duration, years, mean [SD]	8.7 [9.4]	6.6 [7.1]
Active smoker	0 [0%]	3 [10%]
Baseline concomitant 5-ASA dose, g/day [SD]	3.8 [0.8]	3.8 [0.8]
Induction treatment		
Prednisone	10 [34.5%]	12 [40%]
Budesonide	19 [65.5%]	18 [60%]
Disease extension		
E1 Proctitis	3 [10.3%]	3 [10.0%]
E2 Left-sided	19 [65.5%]	17 [56.7%]
E3 Pancolitis	7 [24.1%]	10 [33.3%]
Endoscopic Mayo score at screening		
Mayo 1	1 [3.4%]	1 [3.3%]
Mayo 2	14 [48.3%]	14 [46.7%]
Mayo 3	14 [48.3%]	15 [50.0%]
Faecal calprotectin, mg/kg, median [IQR]	1482 [480–4380]	1920 [273–2871]^*^
C-reactive protein, mg/L, median [IQR]	3.1 [0.8–11.2]	2.6 [1.4–6.7]

^*^One sample was missing.

SD = standard deviation, *n* = number, 5-ASA = 5-aminosalicylates, IQR = interquartile range.

### 3.2 Clinical, endoscopic and histological endpoints

**Figure 3.**
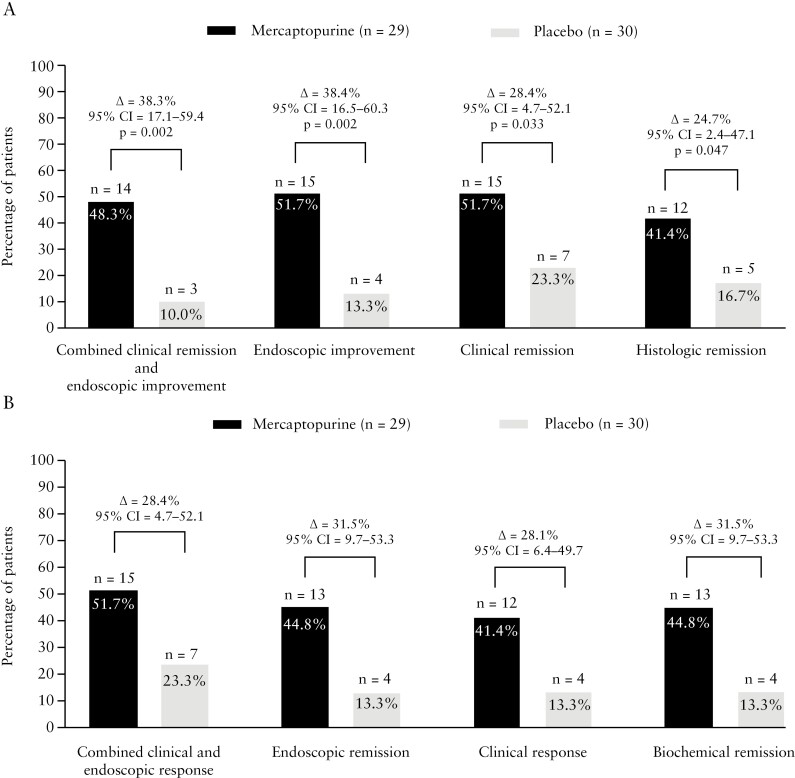
The proportion of patients achieving corticosteroid-free efficacy endpoints at week 52 in an intention-to-treat analysis. [A] The proportion of patients achieving the primary endpoint: corticosteroid-free combined clinical remission and endoscopic improvement [i.e. total score ≤2, and no item >1, using the 12-point Mayo score consisting of stool frequency, rectal bleeding, endoscopic Mayo score and the physician’s global assessment] and the secondary corticosteroid-free endpoints: endoscopic improvement [i.e. endoscopic Mayo score = 0 or 1], clinical remission [i.e. rectal bleeding score = 0 and stool frequency score = 0 or 1 using the 6-point Mayo score with rectal bleeding and stool frequency score] and histological remission [i.e. absence of neutrophils in the mucosa; Geboes score <2 B.1, Robarts histopathology index ≤3 and/or Nancy score ≤1], at week 52 with delta difference, 95% CI of difference and *p*-values. [B] The proportion of patients achieving the remaining corticosteroid-free endpoints: combined clinical and endoscopic response [i.e. 3-point and 30% reduction compared to baseline and 1-point drop in the rectal bleeding score or a rectal bleeding score ≤1], endoscopic remission [endoscopic Mayo score = 0], clinical response (≥2-point drop in the 6-point Mayo score [consisting of rectal bleeding and stool frequency items]) compared to baseline and biochemical remission [CRP <5 mg/L and faecal calprotectin <250 mg/kg] at week 52 with the delta percentage difference between groups with 95% confidence intervals.

In the mercaptopurine group, 16 out of 29 [55.2%] patients continued the study drug up to week 52, compared to 13 out of 30 [43.3%] in the placebo group. At week 52, 14 out of 29 [48.3%] mercaptopurine users achieved the primary endpoint of combined clinical remission and endoscopic improvement compared to three out of 30 [10%] patients in the placebo group (Δ = 38.3%, 95% confidence interval [CI] 17.1–59.4, *p* = 0.002) [Figure 3A]. Of those patients who continued treatment with mercaptopurine up to week 52 [per-protocol analysis], 14 out of 16 [87.5%] reached the primary endpoint compared to three out of 13 [23.1%, Δ = 64.4%, 95% CI 36.4–92.5] in the placebo group at week 52. With regard to the secondary outcomes, the proportions of patients with endoscopic improvement (15/29 [51.7%] vs 4/30 [13.3%], Δ = 38.4%, 95% CI = 16.5–60.3, *p* = 0.002), clinical remission (15/29 [51.7%] vs 7/30 [23.3%], Δ = 28.4%, 95% CI = 4.7–52.1, *p* = 0.033) and histological remission (12/29 [41.4%] vs 5/30 [16.7%], Δ = 24.7, 95% CI 2.4–47.1, *p* = 0.047) at week 52 were significantly larger in the mercaptopurine arm compared to the placebo group. The proportions of patients who attained combined clinical and endoscopic response, endoscopic remission, clinical response and biochemical remission are depicted in Figure 3B. The proportion of patients with a UCEIS of 0, ≤1 and ≤2 at week 52 was numerically higher in the mercaptopurine group compared to the placebo group [Supplementary Table 1].

At week 12, three patients in the mercaptopurine arm and two patients in the thiopurine group did not succeed in tapering down corticosteroids. None of the patients using corticosteroids beyond week 12 reached the primary endpoint at week 52.

### 3.3 Safety

Six out of 29 [20.7%] patients discontinued mercaptopurine due to adverse events that were considered to be related to the study drug: four patients stopped mercaptopurine treatment due to nausea, one due to hepatotoxicity and one due to arthralgia. One patient discontinued mercaptopurine due to an adverse event [i.e. hospitalization due to corticosteroid-induced myopathy], which was not considered to be related to mercaptopurine treatment. Placebo was discontinued by two out of 30 [6.7%] patients due to adverse events that were considered to be related to the study drug: one patient due to palpitations and the other patient due to a skin rash.

**Figure 4.**
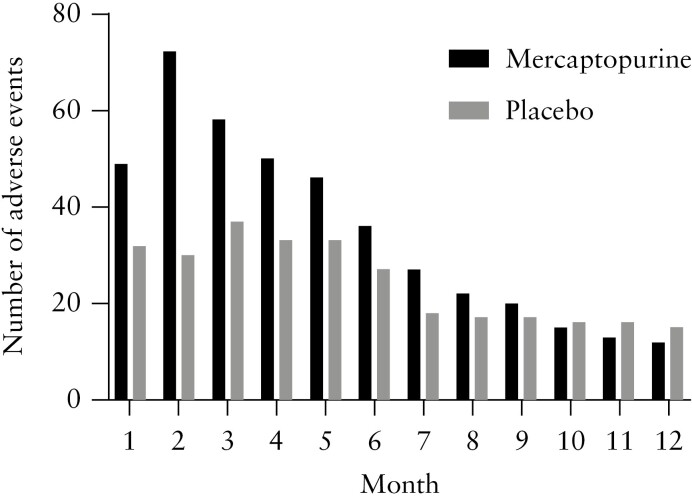
Prevalence of adverse events in the mercaptopurine and placebo group per month. The number on the *x*-axis corresponds to the number of the month in which adverse events occurred.

Except for one patient in the placebo group, all patients experienced at least one adverse event during the study [Table 2]. Patients in the mercaptopurine arm reported 165 adverse events [808.8 per 100 patient-years] compared to 101 [501.4 per 100 patient-years] in the placebo group. Of those adverse events, 82 [49.7%] were likely to be related to the study drug in the mercaptopurine group compared to ten [9.9%] in the placebo group. The prevalence of adverse events per month in the two treatment groups is visualized in Figure 4. More adverse events occurred in the first 6 months in the mercaptopurine group. The prevalence of adverse events was similar between the two treatment groups in the months thereafter. One mercaptopurine-related adverse event was classified as severe: i.e. decrease in leukocyte count to 1.5 × 10^9^/L, which recovered after TDM-based dose adjustment. The most common adverse event was bone marrow suppression in the mercaptopurine arm [35/165, 21.2%] and worsening of UC in the placebo group [23/101, 22.8%]. 5-ASA dose intensification [rectal or oral] was performed in eight [27.6%] patients in the mercaptopurine arm, of whom five [62.5%] reached the primary endpoint compared to nine out of 21 [52.9%, *p* = 0.427] patients without 5-ASA dose intensification in the mercaptopurine group. Seven out of 30 [23.3%] patients required 5-ASA dose escalation in the placebo group, of whom one [14.3%] patient reached the primary endpoint compared to two out of 23 [8.7%, *p* = 1.000] without 5-ASA dose escalation. No drug-induced pancreatitis was observed. The incidence of infections was similar between the two groups. No severe infections occurred and nasopharyngitis was the most commonly observed infection. Four serious adverse events occurred in the mercaptopurine group. Two patients were hospitalized due to acute severe colitis that occurred within the first 4 weeks after initiation of mercaptopurine treatment. One patient was hospitalized with a corticosteroid-induced myopathy and one patient underwent incision and drainage of a perianal abscess without other findings that might fit with Crohn’s disease. One serious adverse event occurred in the placebo group, i.e. hospitalization due to acute severe UC, occurring ~2 months after starting placebo. None of these serious adverse events were considered to be related to the study drug.

**Table 2.** Safety data

	Mercaptopurine [*n* = 29]			Placebo [*n* = 30]		
	*n*	%	Per 100 py	*n*	%	Per 100 py
Total patient-years	20.4			20.1		
Patients with an adverse event	29	100		29	96.7	
Adverse event	165	100	808.8	101	100	501.4
Mild	123	74.5	602.9	63	62.4	312.8
Moderate	36	21.8	176.5	36	35.6	178.7
Severe	6	3.6	29.4	2	2.0	9.9
Adverse event related to study drug	82	49.7	402.0	10	9.9	49.6
Mild	57	34.5	279.4	7	6.9	34.8
Moderate	24	14.5	117.6	3	4.8	14.9
Severe	1	0.6	4.9	0	0.0	0.0
Serious adverse event	4	2.4	19.6	1	1.0	5.0
Serious adverse event related to study drug	0	0.0	0.0	0	0.0	0.0
Adverse events leading to study drug discontinuation	7	4.2	34.3	2	2.0	9.9
Most frequent adverse events [more than 2.5% of total]						
Worsening of ulcerative colitis	18	10.9	88.2	23	22.8	114.2
Bone marrow suppression^*^	26	15.8	127.5	3	3.0	14.9
Nausea	21	12.7	102.9	2	2.0	9.9
Abnormal liver function tests^†^	15	9.1	73.5	4	4.0	19.9
Anaemia	10	6.1	49.0	5	5.0	24.9
Nasopharyngitis	6	3.6	29.4	6	5.9	29.8
Arthralgia	6	3.6	29.4	5	5.0	24.8
Skin lesion	4	2.4	19.6	6	5.9	29.8
Headache	5	3.0	24.5	3	3.0	14.9
Fatigue	3	1.8	14.7	4	4.0	19.9
Infection	18	100	88.2	16	100	79.4
Mild	15	83.3	73.5	15	93.8	74.5
Moderate	3	16.7	14.7	1	6.3	5.0
Severe	0	0.0	0.0	0	0.0	0.0
Type of infection						
Nasopharyngitis	6	33.3	29.4	6	37.5	29.8
Skin infection	3	16.7	14.7	2	12.5	9.9
Flu-like symptoms	2	11.1	9.8	1	6.3	5.0
Conjunctivitis	0	0.0	0.0	2	12.5	9.9
Gastroenteritis	0	0.0	0.0	2	12.5	9.9
Covid-19	1	5.6	4.9	1	6.3	5.0
Urinary tract infection	1	5.6	4.9	1	6.3	5.0
Herpes simplex	2	11.1	9.8	0	0.0	0.0
Herpes zoster	0	0.0	0.0	1	6.3	5.0
Gingivitis [after molar extraction]	1	5.6	4.9	0	0.0	0.0
Otitis	1	5.6	4.9	0	0.0	0.0
Prostatitis	1	5.6	4.9	0	0.0	0.0

*n* = number of events, py = patient years.

^*^Bone marrow suppression included: leukopaenia, lymphopaenia and thrombocytopaenia.

^†^Abnormal liver function tests included: elevation of transaminases, alkaline phosphatase and/or gamma-glutamyltransferase and hypoalbuminaemia. Percentages may not sum to 100% due to rounding

### 3.4 Therapeutic drug monitoring

**Figure 5.**
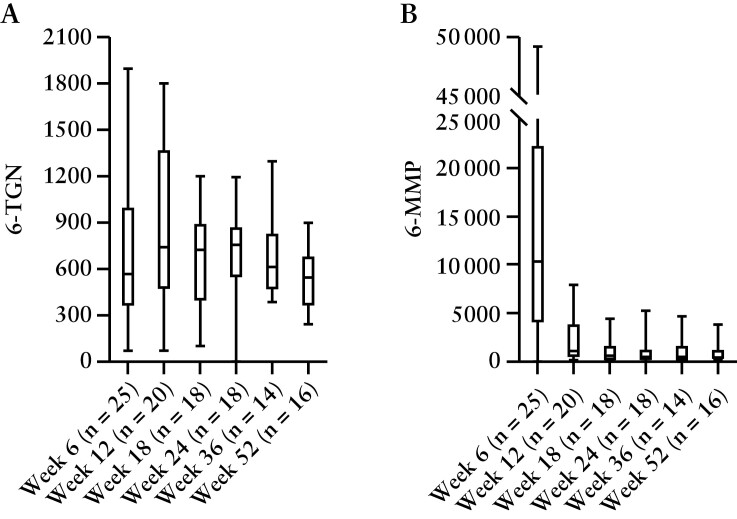
Median 6-thioguaninenucleotide [6-TGN] and 6-methylmercaptopurine [6-MMP] serum concentrations in pmol/8 × 10^8^ red blood cells [RBC] per study visit for patients in the mercaptopurine group who underwent sampling. Boxplots represent interquartile ranges.

Mercaptopurine dose adjustments were required in 22 out of 29 [75.9%] patients. Out of seven patients in the mercaptopurine group who did not require dose adjustments, five [17.2%] discontinued mercaptopurine treatment before the first TDM measurement at week 6, and the other two [6.9%] patients continued mercaptopurine treatment at the initial dose throughout the study up to week 52 [Supplementary Figure 3]. In the placebo group, 12 out of 30 [40%] patients received a dose adjustment, and one [3.3%] patient discontinued placebo treatment before the first TDM measurement at week 6. Seventeen out of 30 [56.7%] patients remained on the initial placebo dose, of whom seven completed the trial and ten participants had to withdraw from the trial early. After the first TDM measurement at week 6, a reduction of the mercaptopurine dose combined with 100 mg allopurinol was required in 14 out of 29 [48.3%] patients. All of them had a 6-MMP concentration exceeding 5700 pmol/8 × 10^8^ RBC. The daily dose of the allopurinol users had decreased from 100 mg/day (interquartile range [IQR] 87.5–125) at enrolment to 25 mg/day [IQR 18.75–25] at week 52 [*p* = 0.007]. Seven other patients in the mercaptopurine group reached week 52 without using allopurinol. By applying TDM, the median initial mercaptopurine dose was decreased from 100 mg/day [IQR 75–100] at enrolment to 50 mg/day [IQR 25–100] at week 52 [*p* = 0.041]. Six out of 15 [40%] patients in the mercaptopurine group, without using allopurinol co-medication, reached the primary endpoint, compared to eight out of 14 [57.1%, *p* = 0.466] patients who started allopurinol co-treatment during the trial. 6-TGN RBC concentrations were stable over time [Figure 5]. A peak in 6-MMP concentration at week 6 was observed, which stabilized after TDM dose adjustments.

## 4. Discussion

We show here that mercaptopurine is superior to placebo in achieving [combined] clinical remission and endoscopic improvement, as well as histological remission at 1 year in UC patients following remission induction treatment with corticosteroids. In total, 55% of patients in the mercaptopurine group completed the trial. Intriguingly, 87.5% of these patients attained combined clinical remission and endoscopic improvement. The majority of patients required TDM-based dosing advice, resulting in a decreased mercaptopurine dose. We therefore conclude that mercaptopurine is a valuable treatment option for UC patients who tolerate it.

Previous randomized controlled trials investigating thiopurine treatment in UC reported clinical and endoscopic remission rates varying between 40 and 76%, which is in line with 48% of patients reaching the primary endpoint in our study.^8–13^ However, endpoint definitions, follow-up periods and patient cohorts varied considerably between these studies and OPTIC. The largest retrospective cohort study that has been performed so far showed that thiopurines were effective in 53% of UC patients, with 20% discontinuing due to intolerance.^25^ A cohort study with nearly complete coverage of an inflammatory bowel disease population in a Dutch province reported a treatment continuation rate of 64% of UC patients who initiated thiopurines.^26^ By way of further comparison with step-up treatments, an earlier study [ACT-1] with infliximab in biologic-naïve UC patients used comparable endpoints but included ~50% thiopurine and 60% corticosteroid non-responders.^27^ Combined clinical remission and endoscopic improvement at 1 year was observed in 35% of patients receiving infliximab. In LOVE-UC, a population of biologic- and immunomodulator-naïve UC patients were treated with vedolizumab, an α4β7-integrin inhibitor. The primary endpoint [similar definition as in OPTIC] was reached by 42% of patients.^28^ Hence, we believe that our observations can abate scepticism regarding the efficacy of thiopurines for the treatment of UC. This is not only relevant for the Western world, but also for developing countries, where the incidence of UC is rising and therapeutic options are often limited.^29,30^ On the other hand, TDM might not always be possible in these countries. Dosing based on MCV, haematology counts and liver function tests may offer a valid alternative to TDM.^31^

Thiopurine-related adverse events hamper their use in daily practice. We observed more drug-related adverse events in the mercaptopurine group and TDM did not prevent these adverse events. The prevalence of adverse events was comparable between the two treatment groups in the second 6 months of the study. For 6-TGN and 6-MMP assays, we applied the Dervieux method with a 6-TGN target of 600–1200 pmol/8 × 10^8^. Dervieux method results are ~2.6 times higher as compared to the Lennard method, which is applied in most clinical studies.^24,32^ This multiplication factor is derived from local analyses at Dutch laboratories and previous literature comparing the two methods.^14,33^ By applying TDM, patients in the mercaptopurine group were often instructed to use a lower dose than the starting dose. Therefore, we believe that a starting dose of 1–1.5 mg/kg might be too high. Treatment initiation with a lower thiopurine dose might blunt an initial 6-MMP peak and thereby reduce dose-dependent adverse events, such as myelotoxicity and hepatotoxicity.^15^ In a previous study, high 6-TGN and 6-MMP levels 1 week after thiopurine initiation were predictive of adverse events, such as nausea and vomiting.^34,35^ In our study, five patients discontinued mercaptopurine before the first metabolite measurement at week 6. It may therefore be advised to perform TDM earlier than 6 weeks after thiopurine treatment initiation. We did not measure TPMT enzyme activity or genotype prior to treatment initiation since it has been shown that TPMT activity is not the main reason for high 6-MMP production.^36^ Measuring TPMT activity would therefore only have given us a partial prediction of the risk of skewed metabolism. Moreover, we did not assess NUDT-15 gene variations, which are associated with thiopurine-induced leukopaenia, as this mutation is rarely observed in our population.^37^ However, starting a lower thiopurine dose in patients with TPMT or NUDT-15 gene variations has been described as a useful strategy to reduce the risk of adverse events.^38,39^ In the study by van Egmond *et al*., only 20% of patients were skewed metabolizers, defined as a 6-MMP/6-TGN ratio of 20.^36^ In our study, skewed metabolism was defined as a 6-MMP concentration >5700 pmol/8 × 10^8^ or a 6-MMP/6-TGN ratio >10 combined with 6-TGN concentration <300 pmol/8 × 10^8^. Thus, we used a lower threshold for skewed metabolism and observed that nearly half of the mercaptopurine users had to start allopurinol co-medication. The proportion of patients on allopurinol–mercaptopurine combination treatment who reached the primary endpoint was numerically higher compared to patients receiving mercaptopurine monotherapy [57 vs 40%, respectively]. This difference was non-significant, which may be due to the relatively small sample size. First-line azathioprine and allopurinol combination therapy was previously shown to be more effective than azathioprine monotherapy.^9,18,40^ Initiating a thiopurine combined with allopurinol or early co-medication with allopurinol, based on 6-MMP and 6-MMP/6-TGN ratio in the first couple of weeks after starting mercaptopurine, may be an attractive treatment strategy.

Our study has several strengths. This was the first prospective controlled study using TDM-based dosing of thiopurine treatment in UC applying random placebo dose adjustments to mimic mercaptopurine treatment in the placebo arm. Second, we used objective endpoints, including endoscopic and histological outcome measures that were assessed in a blinded fashion by experienced readers. Histological remission is today considered an additional treatment target in UC, since it seems to be associated with long-term remission and prevention of colorectal cancer.^41,42^ To our knowledge, OPTIC is the first trial demonstrating superiority of mercaptopurine treatment over placebo in attaining histological remission in UC.

The main limitation of this study was that we did not reach the calculated sample size. Recruitment of patients was challenging because patients sometimes did not accept the risk of taking a placebo or they were unwilling to upscale treatment with a thiopurine. In addition, the coronavirus pandemic significantly interfered with patient recruitment in all clinical trials. Notwithstanding this, and despite the relatively small sample size, our results showed a significant beneficial effect of mercaptopurine using clinical, endoscopic and histological outcome measures. The relatively small sample size did, however, cause large confidence intervals of the differences in the proportion of patients who reached the primary and secondary endpoints. Second, although we conducted a double-blind study, nearly half of the mercaptopurine-treated patients were unblinded as allopurinol co-medication was started. Physicians could also see laboratory results such as MCV and leukocyte counts, which could reveal the treatment allocation. Ideally, the physician would have been blinded to laboratory results and patients in the placebo group would receive a placebo to mimic allopurinol co-medication. However, this might have jeopardized patient safety and an extra placebo would make this investigator-initiated study even more complex to perform. Third, a flare visit was done at the physician’s discretion and the criteria to subsequently withdraw a patient from the study due to inefficacy were not predefined. Nevertheless, study withdrawal was always decided in consultation with the study team and based on endoscopy. Fourth, rectal and oral 5-ASA dose escalation was allowed during the study. However, in a normal clinical setting, 5-ASA treatment should also be optimized in case of a disease flare. Moreover, it was considered unethical to discontinue 5-ASA treatment for patients in the placebo group. Lastly, the control group received placebo instead of weight-based mercaptopurine dosing. The main goal of this placebo-controlled study was to investigate efficacy of thiopurines dosed with an optimal strategy for UC. Since most clinical trials are performed with a placebo-treated control group, patients in the control arm were treated with placebo rather than with weight-based mercaptopurine. Of note, weight-based mercaptopurine treatment in the control group would have increased the required sample size.

In conclusion, this study has demonstrated that proactive TDM-dosed mercaptopurine treatment is more effective than placebo in achieving clinical, endoscopic and histological outcomes in UC patients who failed 5-ASA treatment and received remission induction treatment with corticosteroids. Thiopurines remain a valid treatment option in the emerging therapeutic landscape of UC. However, more adverse events occurred in the mercaptopurine group despite applying TDM. We recommend using lower starting doses of mercaptopurine and to perform early TDM in order to reduce intolerance.

## Supplementary Material

jjad022_suppl_Supplementary_Appendix
