# Supplementary material for: Mercaptopurine for the Treatment of Ulcerative Colitis: A Randomized Placebo-Controlled Trial
Source: J Crohns Colitis. 2023 Feb 27;17(7):1055–65. doi: 10.1093/ecco-jcc/jjad022 (PMC10394500; doi:10.1093/ecco-jcc/jjad022)
Supplement: jjad022_suppl_Supplementary_Appendix [file jjad022_suppl_supplementary_appendix.docx]

**APPENDIX**

**Supplementary material**

| **Supplementary table 1.** Proportions of patients reaching corticosteroid-free endoscopic endpoints using the ulcerative colitis index of severity (UCEIS) | | | | |
| --- | --- | --- | --- | --- |
|  | Mercaptopurine (n=29) | Placebo (n=30) | Δ | 95% CI |
| UCEIS = 0, n (%) | 13 (44.8%) | 4 (13.3%) | 31.5% | 9.7-53.3 |
| UCEIS ≤ 1, n (%) | 14 (48.3%) | 4 (13.3%) | 35.0% | 13.1-56.8 |
| UCEIS ≤ 2, n (%) | 15 (51.7%) | 6 (30.0%) | 21.7% | 8.6-54.9 |

**Supplementary figure 1.** Flowchart TDM based on thiopurine metabolites. 6-TGN and 6-MMP concentrations were measured with the Dervieux method, depicted in units x10^8^ red blood cells (RBC). 6-TGN = 6-thioguaninenucleotides, 6-MMP = 6-methylmercaptopurine.

**Supplementary figure 2.** Flowchart dose adjustments based on adverse events. *Symptoms such as fatigue, nausea/vomiting, pain in the right abdominal upper quadrant, rash, fever and/or eosinophilia. TDM = therapeutic drug monitoring, ULN = upper limit of normal, AST = aspartate aminotransferase, ALT = alanine transaminase, INR = international normalised ratio.

50 mg n = 1 (3%)

75 mg n = 6 (21%)

100 mg n = 14 (48%)

125 mg n = 8 (28%)

25 mg n = 2 (7%)

50 mg n = 3 (10%)

100 mg n = 2 (7%)

12.5 mg with allopurinol n = 2 (7%)*

25 mg with allopurinol n = 6 (21%)

50 mg with allopurinol n = 1 (3%)

Discontinued n = 13 (45%)

**Mercaptopurine daily dose at enrolment Total n = 29 Mercaptopurine daily dose at week 52**

1

1

2

3

1

1

2

3

7

1

3

1

3

1

1

1

1

1

2

2

3

3

1

3

7

3

**Supplementary figure 4.** Sankey diagram of daily dose of mercaptopurine at enrolment and at week 52 (representing patients in the mercaptopurine group). *Patients using 25 mg mercaptopurine every other day together with 100 mg allopurinol every other day.
